# Alzheimer’s Disease and Rheumatoid Arthritis: A Mendelian Randomization Study

**DOI:** 10.3389/fnins.2018.00627

**Published:** 2018-09-12

**Authors:** Qixuan Cai, Zhuoyuan Xin, Lin Zuo, Fan Li, Bin Liu

**Affiliations:** ^1^Key Laboratory of Zoonosis Research, Ministry of Education, College of Basic Medical Science, Jilin University, Changchun, China; ^2^Department of Anesthesiology, China-Japan Union Hospital of Jilin University, Changchun, China; ^3^Center of Cardiovascular Disease, The First Hospital of Jilin University, Changchun, China

**Keywords:** Alzheimer’s disease, rheumatoid arthritis, genome-wide association study, Mendelian randomization, autoimmune disease

## Abstract

Alzheimer’s disease (AD) is the most common neurodegenerative disease. In recent years, multiple pathway analyses of AD genome-wide association studies (GWAS) have been conducted, and provided strong support for immune pathways in AD. Rheumatoid arthritis (RA) is a chronic autoimmune disease. It is reported that antirheumatic drugs had protective effect on dementia in RA patients. However, observational studies have reported a controversial inverse relationship between AD and RA. In addition, Mendelian randomization studies have also been performed to evaluate the association of RA with AD. However, these studies reported inconsistent association of RA with AD. Until now, it is still unclear that AD is a causally associated with RA. Here, we performed a Mendelian randomization study to investigate the causal association of AD with RA. We analyzed the large-scale AD GWAS dataset (74,046 individuals) and RA GWAS dataset (58,284 individuals) from the European descent. However, we did not identify any significant association of AD with RA using inverse-variance weighted meta-analysis (IVW), weighted median regression and MR-Egger regression.

## Introduction

Alzheimer’s disease (AD) is the most common neurodegenerative disease in the elderly (Hu et al., 2017; Liu et al., 2017a, 2018). Until now, it is still largely unknown about the exact AD genes (Jiang et al., 2017). In recent years, multiple large-scale genome-wide association studies (GWAS) have been performed, and successfully identified common AD genes including CR1, BIN1, CLU, PICALM, MS4A4/MS4A6E, CD2AP, CD33, EPHA1, ABCA7, SORL1, HLA-DRB5/DRB1, PTK2B, SLC24A4-0RING3, DSG2, INPP5D, MEF2C, NME8, ZCWPW1, CELF1, FERMT2, and CASS4 (Harold et al., 2009; Hollingworth et al., 2011; Naj et al., 2011; Lambert et al., 2013; Liu et al., 2014b; Li et al., 2016; Jun et al., 2017; Sims et al., 2017). Importantly, some of these genes have been successfully validated (Liu et al., 2012, 2013b,c,d, 2014a,b,c, 2015, 2017a,b; Lambert et al., 2013; Bao et al., 2015; Chen et al., 2015; Li et al., 2015; Shen et al., 2015; Xiang et al., 2015; Zhang et al., 2015; Li et al., 2016; Liu and Jiang, 2016; Zhang et al., 2016; Jiang et al., 2017; Jun et al., 2017; Sims et al., 2017). In addition, multiple pathway analyses of AD GWAS have been conducted, and provided strong support for immune pathways in AD (Hong et al., 2010; Jones et al., 2010; Lambert et al., 2010; Liu et al., 2012). Yokoyama et al. (2016) performed a genetic association study to evaluate the genetic overlap between AD and seven immune-mediated diseases including Crohn’s disease, ulcerative colitis, rheumatoid arthritis (RA), type 1 diabetes, celiac disease, and psoriasis (Jiang et al., 2016; Yokoyama et al., 2016). They identified eight genetic variants associated with both AD and immune-mediated diseases (Jiang et al., 2016; Yokoyama et al., 2016). However, epidemiological studies have reported a controversial inverse relationship between AD and RA (Ferraccioli et al., 2012; Kao et al., 2016; Ungprasert et al., 2016).

Mendelian randomization could determine the causal inferences, and has been used to evaluate the association between RA and AD (Policicchio et al., 2017; Bae and Lee, 2018). Policicchio et al. (2017) selected 62 RA SNPs (*P* < 5.00E-08, a genome-wide significance level) as instrumental variables, and identified no evidence of a causal association between RA and AD. Bae and Lee (2018) selected 80 RA SNPs as instrumental variables. They selected three methods including IVW, weighted median, and MR-Egger (Bae and Lee, 2018). Both the IVW (beta = −0.039, *P* = 0.021) and weighted median (beta = −0.078, *P* = 0.001) indicated significant association of RA with AD (Bae and Lee, 2018). In summary, both studies evaluated the causal association of RA with AD, and reported inconsistent findings (Policicchio et al., 2017; Bae and Lee, 2018). Importantly, both studies did not evaluate the causal association of AD with RA. Until now, it is still unclear whether AD is a causally associated with RA. Here, we performed a Mendelian randomization study to investigate the causal association of AD with RA.

## Materials and Methods

### AD GWAS Dataset

The instrumental variables are AD variants at a genome-wide significance level *P* < 5.00E-08 identified by previous GWAS. The AD GWAS dataset is from the International Genomics of Alzheimer’s Project (IGAP) (Lambert et al., 2013). In stage 1, the IGAP analyzed a total of 17,008 AD cases and 37,154 controls of European descent (The European Alzheimer’s disease Initiative – EADI, the Alzheimer Disease Genetics Consortium – ADGC, The Cohorts for Heart and Aging Research in Genomic Epidemiology consortium – CHARGE, The Genetic and Environmental Risk in AD consortium – GERAD) (Lambert et al., 2013). In stage 2, IGAP analyzed additional independent 8,572 AD cases and 11,312 controls (Lambert et al., 2013). Here, we aimed to selected the independent AD variants at a genome-wide significance level *P* < 5.00E-08 in this AD dataset (Lambert et al., 2013).

### RA GWAS Dataset

The RA GWAS dataset is from a previous RA GWAS meta-analysis in a total of >100,000 subjects of European and Asian ancestries (29,880 RA cases and 73,758 controls) (Okada et al., 2014). The summary statistics of RA GWAS meta-analysis included trans-ethnic RA GWAS meta-analysis (19,234 RA cases and 61,565 controls), European RA GWAS meta-analysis (14,361 RA cases and 43,923 controls), and Asian RA GWAS meta-analysis (4,873 RA cases and 17,642 controls) (Okada et al., 2014). Here, we selected the European RA GWAS meta-analysis, as the AD GWAS dataset was also from European samples.

### Mendelian Randomization Analysis

Here, we selected three Mendelian randomization analysis methods including inverse-variance weighted meta-analysis (IVW), weighted median regression and MR-Egger regression, as did in recent studies (Bae and Lee, 2018; Jiang et al., 2018). In addition, we selected the MR-Egger intercept test to assess the instrumental variable assumptions, and provide a statistical test for the presence of potential pleiotropy (Bae and Lee, 2018; Jiang et al., 2018). The odds ratio (OR) as well as 95% confidence interval (CI) of RA correspond to the genetically determined increase in AD. Meanwhile, we performed a sensitivity analysis using a leave-one-out permutation. All analyses were conducted using the R package “MendelianRandomization” (Yavorska and Burgess, 2017). The significance level for significant association of AD with RA was *P* < 0.05.

## Results

### Association of AD Variants With RA

The meta-analysis of stage 1 and stage 2 in IGAP identified 21 independent AD variants at the genome-wide significance level *P* < 5.00E-08. Of the 21 AD risk variants, we extracted the summary statistics of 20 variants in RA GWAS. Only one variant rs10745742 and its proxy variants with *r*^2^ > = 0.8 in HaploReg v4.1 in 1000 Genomes Project (CEU) (Ward and Kellis, 2012), were not available in RA GWAS dataset. Hence, our analysis will focus on these 20 variants. Here, we provided more detailed information about these 20 variants in **Table 1**.

**Table 1:** Characteristics of 20 genetic variants in RA and AD GWAS datasets.

SNP	Chromosome	Position	Effect allele	Non-effect allele	AD GWAS			RA GWAS		
					Beta	Standard error	*P*-value	Beta	Standard error	*P*-value
rs6656401	1	207692049	A	G	0.1667	0.0165	5.69E-24	−0.0109	0.020625535	0.46
rs6733839	2	127892810	T	C	0.1965	0.0141	6.94E-44	0.0237	0.022413611	0.27
rs35349669	2	234068476	T	C	0.0755	0.0136	3.17E-08	−0.021	0.020836232	0.25
rs190982	5	88223420	G	A	−0.0759	0.0137	3.23E-08	−0.0237	0.022413611	0.26
rs10948363	6	47487762	G	A	0.0954	0.0145	5.20E-11	−0.0044	0.01777549	0.67
rs2718058	7	37841534	G	A	−0.0774	0.0132	4.76E-09	0.0313	0.021051282	0.091
rs1476679	7	100004446	C	T	−0.0891	0.0144	5.58E-10	−0.0381	0.024547967	0.12
rs11771145	7	143110762	A	G	−0.102	0.0137	1.12E-13	−0.0113	0.025789826	0.65
rs28834970	8	27195121	C	T	0.0959	0.0162	3.27E-09	0.0008	0.020419058	0.84
rs9331896	8	27467686	C	T	−0.146	0.0141	2.77E-25	0.0309	0.015784542	0.058
rs10838725	11	47557871	C	T	0.0789	0.0138	1.12E-08	−0.0237	0.022413611	0.34
rs983392	11	59923508	G	A	−0.1081	0.0134	6.14E-16	0.0313	0.021051282	0.15
rs10792832	11	85867875	A	G	−0.14	0.0133	9.32E-26	−0.0005	0.015310717	0.98
rs11218343	11	121435587	C	T	−0.2697	0.041	4.98E-11	0.0152	0.051712121	0.74
rs17125944	14	53400629	C	T	0.1323	0.0229	7.95E-09	−0.0561	0.033741829	0.097
rs10498633	14	92926952	T	G	−0.1044	0.0199	1.47E-07	−0.0264	0.023564622	0.23
rs8093731	18	29088958	T	C	−0.6136	0.1123	4.63E-08	−0.002	0.094043657	0.98
rs4147929	19	1063443	A	G	0.143	0.0178	1.06E-15	−0.0221	0.031276103	0.48
rs3865444	19	51727962	A	C	−0.0667	0.0143	2.97E-06	−0.0109	0.020625535	0.61
rs7274581	20	55018260	C	T	−0.1323	0.0237	2.46E-08	0.0331	0.036882967	0.4

*Beta is the overall estimated effect size for the effect allele, beta = ln(odd ratio); Beta > 0 and Beta < 0 means that this effect allele could increase and reduce disease risk, respectively.*

### Association of AD With RA

In brief, we did not identify any significant association of AD with RA including the IVW (OR = 0.95, 95% CI: 0.88–1.03, *P* = 0.451), weighted median (OR = 0.96, 95% CI: 0.85–1.07, *P* = 0.217), and MR-Egger (OR = 0.98, 95% CI: 0.78–1.22, *P* = 0.827). In addition, MR-Egger intercept test did not show significant pleiotropy (MR-Egger intercept β = −0.003; *P* = 0.804). Hence, the estimates from these methods were consistent in terms of direction and magnitude. The leave-one-out permutation analysis showed that the direction and precision of the genetic estimates between AD and RA remained largely unchanged. **Supplementary Figure S1** shows the individual causal estimates from each of the 20 genetic variants using different methods.

## Discussion

Observational studies have evaluated the association between AD and RA. However, these studies reported inconsistent findings. Chou et al. (2016) conducted a nested case-control study by analyzing more than 8.5 million commercially insured adults. They found that AD was more prevalent among RA patients compared with those without RA (Chou et al., 2016). RA population had an increased AD risk (Chou et al., 2016). Kao et al. (2016) performed a case-control study to evaluate the relationship between prior RA and AD using 2271 patients with AD as cases and 6813 patients without AD as controls. They found an inverse association between prior RA and AD (Kao et al., 2016). Ungprasert et al. (2016) conducted a systematic review and meta-analysis of three cohort studies and two cross-sectional studies. They identified significant increased risk of dementia in RA cases (Ungprasert et al., 2016). Policicchio et al. (2017) a systematic review and meta-analysis of eight case-control and two population-based studies. They found that RA was associated with lower AD incidence (Policicchio et al., 2017).

Genetic association studies also have evaluated the association between AD and immune pathways. These findings are consistent. Several pathway analyses of AD GWAS dataset have identified some immune pathways in AD, including Natural killer cell mediated cytotoxicity (hsa04650), Antigen processing and presentation (hsa04612), retinoic acid-inducible gene-I (RIG-I)-like receptor signaling (hsa04622), asthma (hsa05310), hematopoietic cell lineage (hsa04640), graft-versus-host disease (hsa05332), allograft rejection (hsa05330), autoimmune thyroid disease (hsa05320), and type I diabetes mellitus (hsa04940) (Hong et al., 2010; Jones et al., 2010; Lambert et al., 2010; Liu et al., 2012). Yokoyama et al. (2016) found genetic overlap between AD and immune-mediated diseases (Jiang et al., 2016).

Until now, two Mendelian randomization studies have also been performed to evaluate the association between RA and AD (Policicchio et al., 2017; Bae and Lee, 2018). However, these two studies reported inconsistent findings. Policicchio et al. (2017) reported no evidence of a causal association between RA and AD. Bae and Lee (2018) identified a significant causal association of RA with AD. Judge et al. (2017) found that antirheumatic drugs had protective effect on dementia in RA patients. The classical disease-modifying antirheumatic drug (cDMARDs) users, especially the methotrexate users, had a reduced dementia risk (Judge et al., 2017).

In summary, AD and RA are the most common neurodegenerative disease and a chronic autoimmune disease, respectively (Liu et al., 2013a). Until now, observational studies, genetic association studies, and Mendelian randomization studies have reported inconsistent association between AD and RA. Here, we conducted a Mendelian randomization study to investigate the causal association of AD with RA. We did not identify any significant association of AD with RA.

Our Mendelian randomization study may have several strengths. First, we selected large-scale AD GWAS dataset (74,046 individuals) and RA GWAS dataset (58,284 individuals) from the European descent. This could reduce the influence of the population stratification. Second, the instruments consisted of 20 independent AD genetic variants, which could reduce the influence on of linkage disequilibrium. Third, we selected three Mendelian randomization methods, as did in recent studies.

## Author Contributions

QC and BL designed the study, collected the samples, and clinic information. QC, BL, ZX, LZ, and FL analyzed the data and wrote the paper.

## Conflict of Interest Statement

The authors declare that the research was conducted in the absence of any commercial or financial relationships that could be construed as a potential conflict of interest. The reviewer CW and handling Editor declared their shared affiliation at the time of the review.

## Supplementary Material

FIGURE S1 Causal estimates from single genetic variant using different Mendelian randomization analysis methods.
