# Supplementary figures and images for: Alzheimer’s Disease and Rheumatoid Arthritis: A Mendelian Randomization Study
Source: Front Neurosci. 2018 Sep 12;12:627. doi: 10.3389/fnins.2018.00627 (PMC6143656; doi:10.3389/fnins.2018.00627)

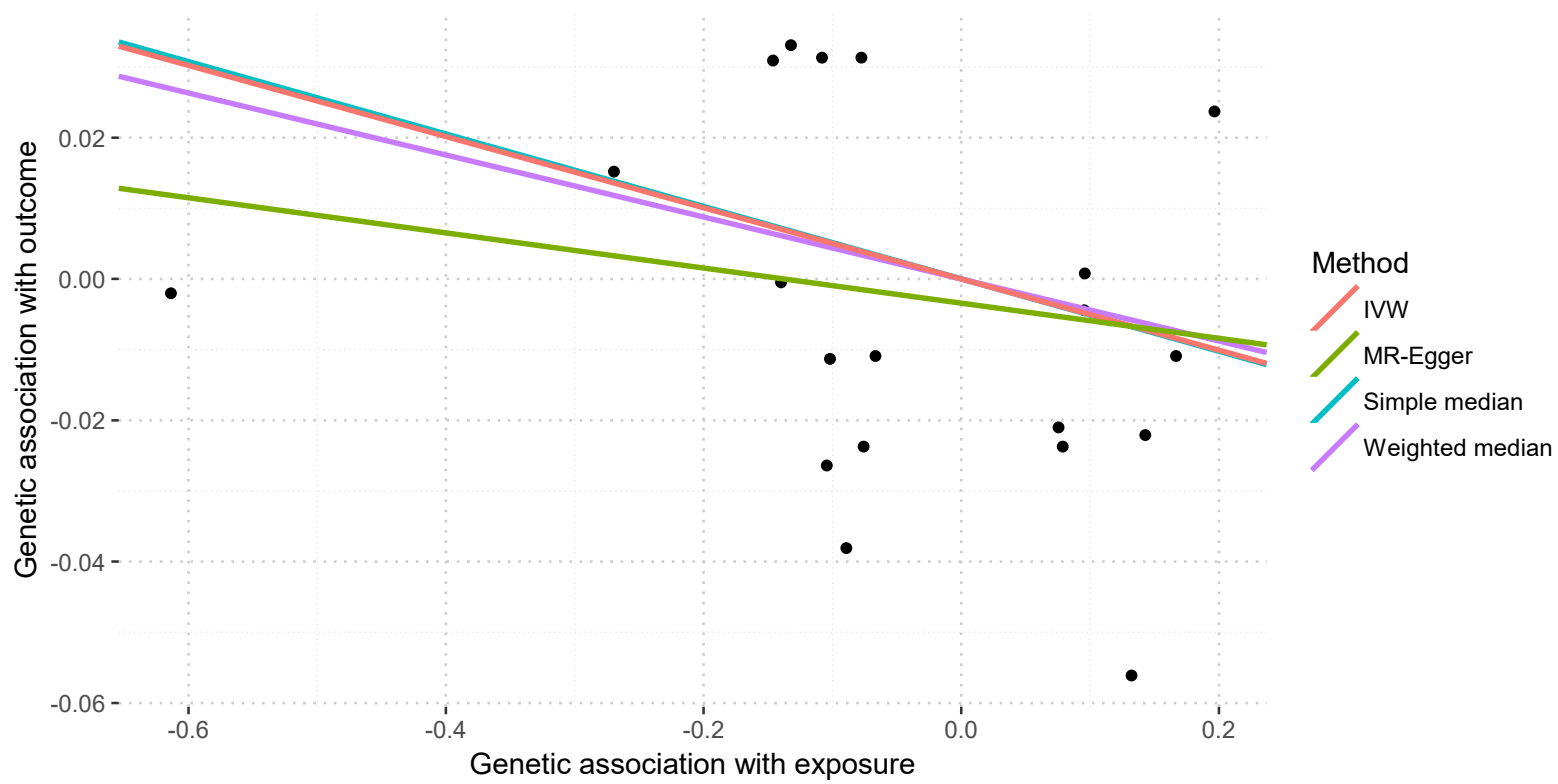

Supplement: FIGURE S1 — Causal estimates from single genetic variant using different Mendelian randomization analysis methods. [file Data_Sheet_1.PDF]
